# Clinical trials on onabotulinumtoxinA for the treatment of chronic migraine

**DOI:** 10.1007/s10194-011-0333-5

**Published:** 2011-03-27

**Authors:** Michael Bjørn Russell

**Affiliations:** 1 Head and Neck Research Group, Research Centre, Akershus University Hospital, 1478 Lørenskog, Oslo, Norway; 2 Institute of Clinical Medicine, University of Oslo, 1474 Nordbyhagen, Oslo, Norway

Migraine is probably the second most common pain disorder next to tension-type headache. The majority of migraineurs have episodic attacks, but a small minority have chronic migraine [1, 2]. Treatment options for chronic migraine are sparse. Thus, clinical trials investigating new treatment options are therefore endorsed in order to provide evidence-based data.

Two double-blind, randomized, placebo-controlled clinical trials for the treatment of chronic migraine with onabotulinumtoxinA, the Phase III REarch Evaluating Migraine Prophylaxis Therapy (PREEMPT) 1 and 2 as well as the pooled data from the two trials were recently published [3–5].

The introduction of the three onabotulinumtoxinA papers describes the definition of chronic migraine according to the criteria of the International Classification of Headache Disorders II (ICHD II) [6, 7].

The PREEMPT inclusion criteria for chronic migraine were:

1. Migraine according to the ICHD II excluding hemiplegic migraine, basilar-type migraine, ophthalmoplegic migraine and migrainous infarction.
2. ≥15 headache days within 28 days at baseline, with each day consisting of ≥4 h of continuous headache and with ≥50% of days being migraine or probable migraine days.

Two of the PREEMPT exclusions criteria were:

1. Diagnosis of other primary or secondary headache disorders.
2. Use of prophylactic medication within 28 days before start of baseline.

At a first glance it seems that chronic migraine in the PREEMPT studies adhere to criteria of the ICHD II, as other primary and secondary headache disorders were excluded. However, the latter was not specified in details and also lacked a reference, although one might get the impression that the criteria of the ICHD II were applied, since this classification is mentioned in the beginning of the study participants section [3, 4]. However, Table 1 in the results section shows that approximately 2/3 of all PREEMPT 1 and 2 included participants with chronic migraine had overused acute headache pain medication at baseline [3, 4]. The pooled data publication do not include this information in Table 1, but the result section mention that most patients overused acute pain medication at baseline and 1.7% had opioid overuse [5]. The mean years since onset of chronic migraine varied between 17.6 and 20.6 years [3, 4]. Thus, it is reasonable to assume that the medication overuse had persisted for at least 3 months in the majority of the included participants with medication overuse at baseline. According to the criteria of the ICHD II chronic migraine is classified only if there is no medication overuse (criteria D) [6, 7]. Thus, the 2/3 of the PREEMPT 1 and 2 included participants with chronic migraine and medication overuse should be classified medication overuse headache and not chronic migraine [6–8]. This leaves only approximately 1/3 of PREEMPT 1 and 2 participants included with a ICHD II diagnosis of chronic migraine. In fact it might be less, since the ICHD II definition of chronic migraine only includes migraine without aura and not migraine with aura. No doubt classification on chronic migraine and medication overuse headache has been a challenge within the last two decades, since the ICHD I from 1988 did not include chronic migraine, and the ICHD II from 2004 was soon revised [6–9]. This has been described in details elsewhere [10]. However, the description of different chronic migraine criteria in different part of the three onabotulinumtoxinA papers are confusing and misleading, especially since medication overuse is not explicit mentioned in the methods section (study participants sections) [3–5].

The heterogeneity of the study participants raise questions in relation to efficacy of onabotulinumtoxinA on the primary and secondary end point defined in the PREEMPT 1 and 2. Firstly, what is the efficacy of onabotulinumtoxinA in those with medication overuse headache (chronic migraine without aura and medication overuse headache)? Secondly, what is the efficacy of onabotulinumtoxinA in those with chronic migraine (chronic migraine without aura without medication overuse)? Furthermore, how do factors such as migraine with aura, gender, race and previously used prophylactic medication influence on the efficacy of onabotulinumtoxinA? Such evidence-based data are warranted for those treating chronic migraine and medication overuse headache.

Data on those whom had medication overuse headache and those whom had previously tried prophylactic medication are especially important. Short information to people with chronic headache (≥15 days/month for at least 3 months) and medication overuse about the possible role of medication overuse in headache chronification, and no further contact before follow-up approximately 1½ years later, reduced mean medication days from 22 to 6 days/month, and 42% no longer had chronic headache [11]. Fifty-three percentage of the participants had co-occurrence of migraine, but only a few had chronic migraine. Since enrollment of the PREEMPT 1 and 2 required that prophylactic medication was not taken 28 days prior to the baseline data collection, it is likely to assume that those enrolled had none or only a modest effect of previous tried prophylactic medications. This is important in the light that 79% (−6.6/−8.4) of the reduction of headache days (primary efficacy variable) was due to placebo response [5]. Hopefully, future publication on onabotulinumtoxinA will enlighten us regarding the efficacy of onabotulinumtoxinA for the treatment of chronic migraine and medication overuse headache among others.
